# Preparation of high-performance supercapacitor electrode with nanocomposite of CuO/NCNO flower-like

**DOI:** 10.1038/s41598-023-43430-1

**Published:** 2023-09-27

**Authors:** Esmail Sohouli, Hakimeh Teymourinia, Ali Ramazani, Koroush Adib

**Affiliations:** 1 Department of Chemistry, Faculty of Science, University of Imam Hossein, Tehran, Iran; 2 Department of Biotechnology, Research Institute of Modern Biological Techniques (RIMBT), University of Zanjan, Zanjan, 45371-38791 Iran; 3 Department of Chemistry, Faculty of Science, University of Zanjan, Zanjan, 45371-38791 Iran

**Keywords:** Nanoscale materials, Other nanotechnology, Chemistry, Materials science, Nanoscience and technology

## Abstract

Due to the importance of energy storage systems based on supercapacitors, various studies have been conducted. In this research CuO, NCNO and the flower like CuO/NCNO have been studied as a novel materials in this field. The resulte showed that the synthesized CuO nanostructutes have flower like morphology which studied by FE-SEM analisis. Further, the XRD pattern confirmed the crystalline properties of the CuO/NCNO nanocomposite, and the Raman verified the functional groups and vibrations of the components of CuO/NCNO nanocomposite. In a two-electrode system at a current density of 4 A/g, the capacitance, power density, and energy density were 450 F/g, 3200 W/kg, and 98 Wh/kg, respectively. The charge transfer resistances of CuO and NCNO/CuO electrodes obtained 8 and 2 Ω respectively, which show that the conductivity and supercapacitive properties of nanocomposite are better than pure components. Also, the stability and low charge transfer resistance are other advantages obtained in a two-symmetrical electrode investigation. The stability investigation showed that after 3000 consecutive cycles, only 4% of the initial capacitance of the CuO/NCNO electrode decreased.

## Introduction

The use of energy storage devices such as batteries and conventional capacitors has been limited due to their large size, low power density, low capacitance, long times required for charging, short lifespan, and environmental pollution^1–9^. Introducing supercapacitors as developed electrochemical capacitors with excellent features including very high density, high speed of charging and discharging, high capacitance, and biocompatibility as well as their industry-scale application (electric vehicles, electrical installations, and the electricity support of factories) and so on have partially resolved the concerns created in the field of using energy storage devices^10–17^. Electrochemical supercapacitors have two mechanisms for charge storage depending on the type of materials used in the electrode structure. The charge storage mechanism at the electrode–electrolyte interface is due to the ion adsorption in an electric double-layer capacitor (EDLC)^18,19^. The EDLC electrochemical supercapacitors system mainly consists of carbon materials with high surface area and porous structures such as activated carbon, carbon nanotubes, and graphene^20–26^. In pseudo-capacitors, the charge storage mechanism is based on redox reactions or electron transfer (faradaic reactions). Conductive polymers, metals, and transition metal oxides are often used in their structure^27–31^. Carbon nanomaterials are the best materials in electrode for electrochemical supercapacitors owing to their easy accessibility, high chemical and mechanical stability, large surface area, and excellent electrical conductivity. Despite the abundant use of pure carbon nanomaterials in electrochemical fields, especially for energy storage, low capacitance, and energy density have limited their commercial use. Doping heteroatoms such as phosphorus (P), nitrogen (N), oxygen (O), sulfur (S), and fluorine (F) in the structure of the carbon matrix is one of the suitable modification approaches for improving the electrochemical properties and commercialization of carbon nanomaterials^32–34^. Nitrogen (N) heteroatom has been studied more than other heteroatoms due to the ease of integration in the structure of carbon nanomaterials, placement in the structure with different chemical forms, and creation of active sites for redox reactions. Also, N atoms, having high electronegativity compared to C and H atoms, induce a positive charge on the adjacent carbon atoms, which increases the active sites on the electrode surface to bind electrolyte ions and enhances electrode wettability^35–37^. Carbon nano onions (CNOs) are carbon particles containing multiple layers of graphene with SP^2^ hybridization^38,39^. The layered structure in CNOs spheres has caused the creation of many ion channels and pores, as well as increasing the accessibility of the outer surface of the structure, and these structural features increase its electrocatalytic activity^40–42^. Despite the good electrochemical activity and high charge–discharge rate, the capacitance and specific energy are low in CNOs structures^43,44^. A basic approach for raising the specific capacitance and the resistance of CNO structures is to dope N atoms in their structure, which can increase active surface centers for electrolyte ions adsorption on its surface, resulting in increased capacitance^43,45^. Based on the few provided reports on the application of N-doped CNOs structures, much effort is needed to improve these carbon structures’ application in the supercapacitors field^46^. Using transition metal oxides (TMOs) in the electrode structure increases reversible redox reactions, energy density, and capacitance^47,48^. Although using TMOs in the electrode structures (pseudo-capacitive electrodes) increases the specific capacitance, they can effectively reduce the energy density due to the limited potential window. The primary and practical solution is the nanocomposite fabricationby combining TMOs as pseudo-capacitor materials with carbon nanomaterials as EDLC materials and applying the metal oxide/carbon nanostructuresbased-nanocomposites as electrode materials in electrochemical supercapacitors^49,50^. The capacitors made by the mentioned nanocomposite materials are a hybrid of EDLC capacitors and pseudo-capacitors that have excellent properties of both types.

The formation of nanocomposite from carbon-based nanomaterials and TMOs is a suitable approach for improving the specific capacitance of CNOs and increasing its applications in electrochemical supercapacitors. Up to now, few studies have been reported on this area. Wang et al. reported Co_3_O_4_ /CNOs composite as electrochemical supercapacitor material^51^. The introduction of CNOs improved the structural stability and conductivity of Co_3_O_4_ electrode material. We designed CNOs/ MnO_2_/Fe_3_O_4_ nanocomposite as supercapacitor electrode materials^52^. Singh et al.^53^ used nanoporous gold (Au)–N doped CNOs-based micro-supercapacitor for developing an energy storage unit compatible with microelectronic devices. Due to favorable properties such as easy and fast synthesis, high abundance, excellent electrochemical and electrocatalytic properties, low toxicity, and variable morphology of nanoparticles, copper oxide (CuO) has been proposed as one of the widely used metal oxides in electrode materials for lithium-ion batteries, electrochemical capacitors and antibacterial^54,55^. Based on the latest information, no study has been done on making nanocomposite from the combination of CuO and N-CNOs and its application in hybrid electrochemical capacitors.

The properties of selected materials play a significant role in the capacitance of supercapacitors and current density, so choosing a suitable material for a supercapacitor is essential. Meanwhile, the type of morphology also causes many differences in the results. Choosing an environmentally friendly material with excellent ability as electrode material in a supercapacitor is necessary. Copper oxide is a good choice among metal oxides due to its reasonable price, good electrochemical properties, and environmental compatibility^56^. However, the electrochemical performance of copper oxide is still far from the amount required for practical applications due to its relatively low electrical conductivity. One of the ways to improve this problem is to use copper oxide-based nanocomposite with special morphology. Therefore, in this work, copper oxide with flower-like morphology is prepared^57^. This morphology prevents accumulation and aggregation in oxide nanoparticles and increases the electron transfer rate between the electrode and the electrolyte. In addition, considering the high surface area obtained from copper oxide in this morphology, more ions in electrolyte diffuse to the surface, resulting in the increased supercapacitor performance and properties of the oxide^58–60^. The surface area of copper oxide is improved by the presence of NCNO, which has good conductivity. Therefore, a new and desirable nanocomposite is prepared for designing a supercapacitor with high performance, increasing the stability of copper oxide in alkaline solutions, and bringing it closer to practical applications. According to the literature, CuO/NCNO nanocomposite with flower-like morphology has not been prepared so far. On the other hand, in this work, CuO/NCNO nanocomposite is synthesized directly on the nickel foam surface without using any polymer additives that affect the conductivity of the nanocomposite, as a result, both time and cost of consumables for supercapacitor preparation are saved. So, In this work, copper oxide nanoparticles were used to form a nanocomposite with N-doped CNO nanoparticles for use as electrode materials in electrochemical supercapacitors. The structure of NCNOs/CuO nanocomposite was characterized using SEM and XRD techniques. Cyclic voltammetry (CV), electrochemical impedance spectroscopy (EIS), and charge–discharge methods were used for the electrochemical analysis of the proposed nanocomposite-based supercapacitors.

## Experimental

### Materials and characterization methods

Chitosan (Medium molecular weight, 98.0%), copper sulfate pentahydrate (99%), acetic acid (100%), potassium hydroxide (99%), hexamethylenetetramine (HMTA, 99%), were purchased from Merck company. The microstructure and distribution of elements of the catalysts were investigated by Field Emission Scanning Electron Microscopy (FE-SEM, TESCAN MIRA 3) outfitted with an energy dispersive spectrometer (EDS). X-ray diffraction (XRD) patterns were recorded using a Bruker D8 Advance diffractometer with Cu Kα radiation.

### NCNO synthesis

NCNO was prepared from a low-price material without using hazardous materials. For this purpose, 1 g of chitosan was mixed with 10% acetic acid at a temperature of 60 °C under stirring. Then 1 g of chitosan gel was mixed with 25 mL of water and introdused into the autoclave and put in an oven at 180 °C for 8 h. Finally, after the autoclave cooled down, the black product was washed with water and ethanol and dried at 60 °C.

### CuO/NCNO flower- like synthesis

The hydrothermal method was used to synthesize of flower-like CuO. For this mean, 1 g of copper sulfate was dissolved in 50 ml of water and stirred for 30 min. Also, 2 gr of HMTA was dissolved in 25 mL of water and added dropwise to the solution containing copper sulfate and stirred for 30 min. Afterward, the reaction mixture was transferred to an autoclave and heated at 180 °C for 10 h. After completing the reaction and cooling the product to ambient temperature, the flower-like CuO black product was separated by centrifuging, washing with water and ethanol, and dried at 60 °C for 12 h. The same method was used to synthesize the flower-like CuO/NCNO nanocomposite. During the pripartion of composite, 50 mg of NCNO was also exist in the solution. Also, after washing with acetone and water and drying at 60 °C for 12 h, nickel foam (1 × 1 cm) was placed in the autoclave, including nanocomposite precursors. After obtaining the final product, the nickel foam containing the product was washed with water and dried in an oven.

To obtain the specific capacitance (SC) of three- and two-electrode systems, Eqs. (1) and (2) are used, respectively. Also, Eqs. (3) and (4) are used to calculate power and energy density.

1 $${\text{SC }} = \frac{I \cdot t}{{m\Delta E}}$$

2 $${\text{SC }} = { 2}\frac{I \cdot t}{{m\Delta E}}$$

3 $${\text{E }} = \frac{{C \cdot V^{2} }}{2}$$

4 $${\text{P }} = {\text{ E}}/{\text{t}}$$

## Result and discussion

### Characterization

**Figure 1:**
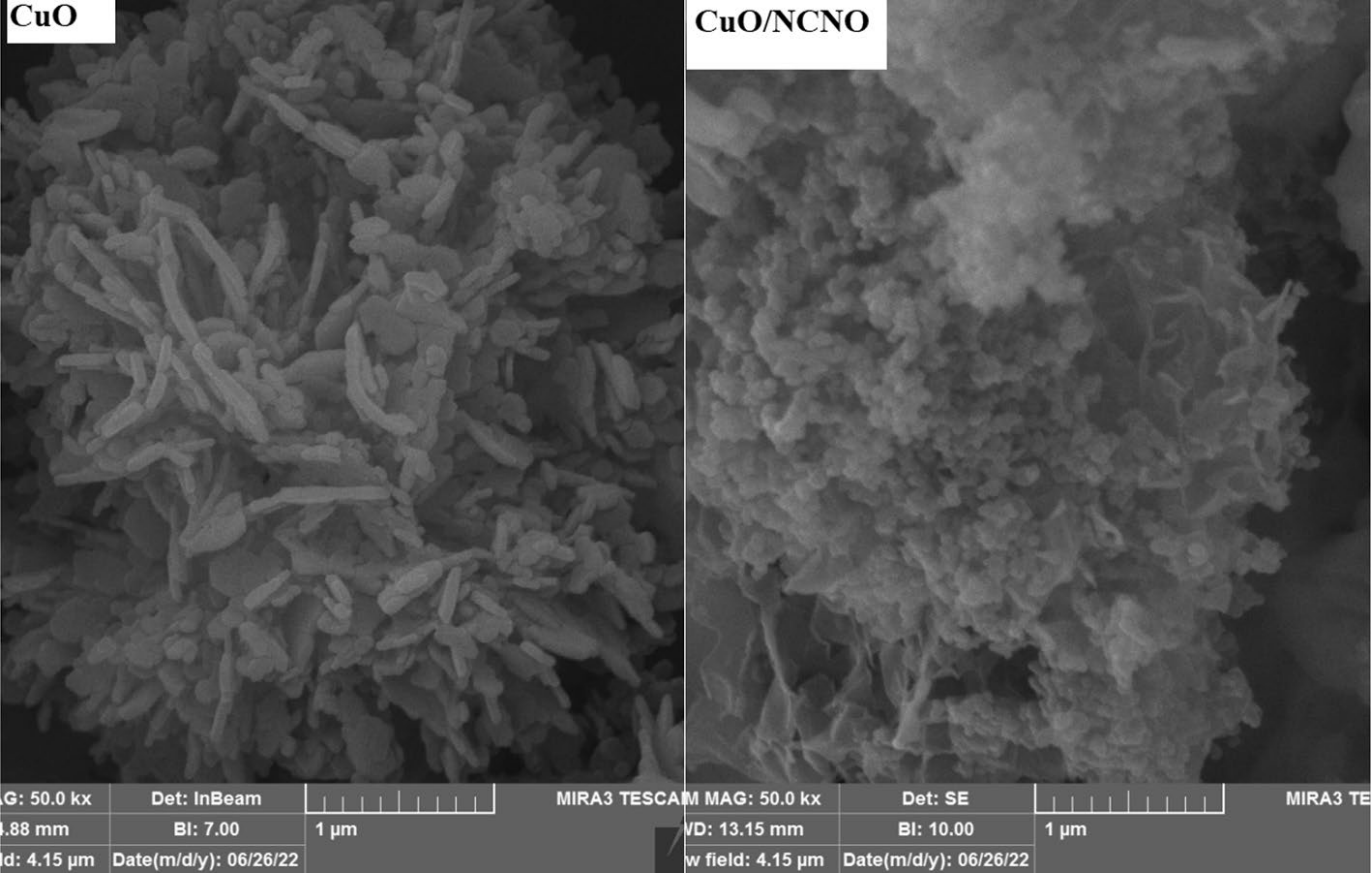
FE-SEM image of CuO and CuO/NCNO.

FE-SEM images of flower-like CuO and CuO/NCNO are shown in Fig. 1. As the FE-SEM images of copper oxide show, a flower-like morphology can be observed for this nanostructure. The same flower-like structure can be observed in the presence of NCNO spherical nanoparticles.

**Figure 2:**
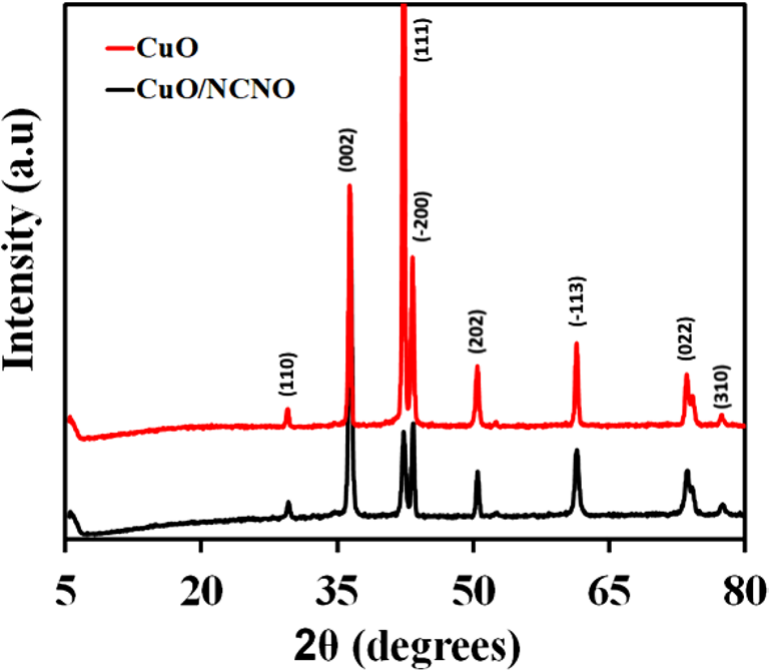
XRD pattrn of CuO and CuO/NCNO.

The XRD patterns of flower-like CuO and CuO/NCNO are shown in Fig. 2. The peaks observed at 2θ = 30.7, 36.9, 42.8, 43.7, 50.6, 61.6, 74.3, and 77 degrees are related to the (110), (002), (111), (− 200), (202), (− 113), (022) and (310) planes, respectively (according to JCPDS 0661-05) [95]. The same peaks can also be seen in the CuO/NCNO nanocomposite; however, the peak related to NCNO is not observed in the XRD pattern of the composite due to the low amounts of NCNO compared to CuO.

**Figure. 3:**
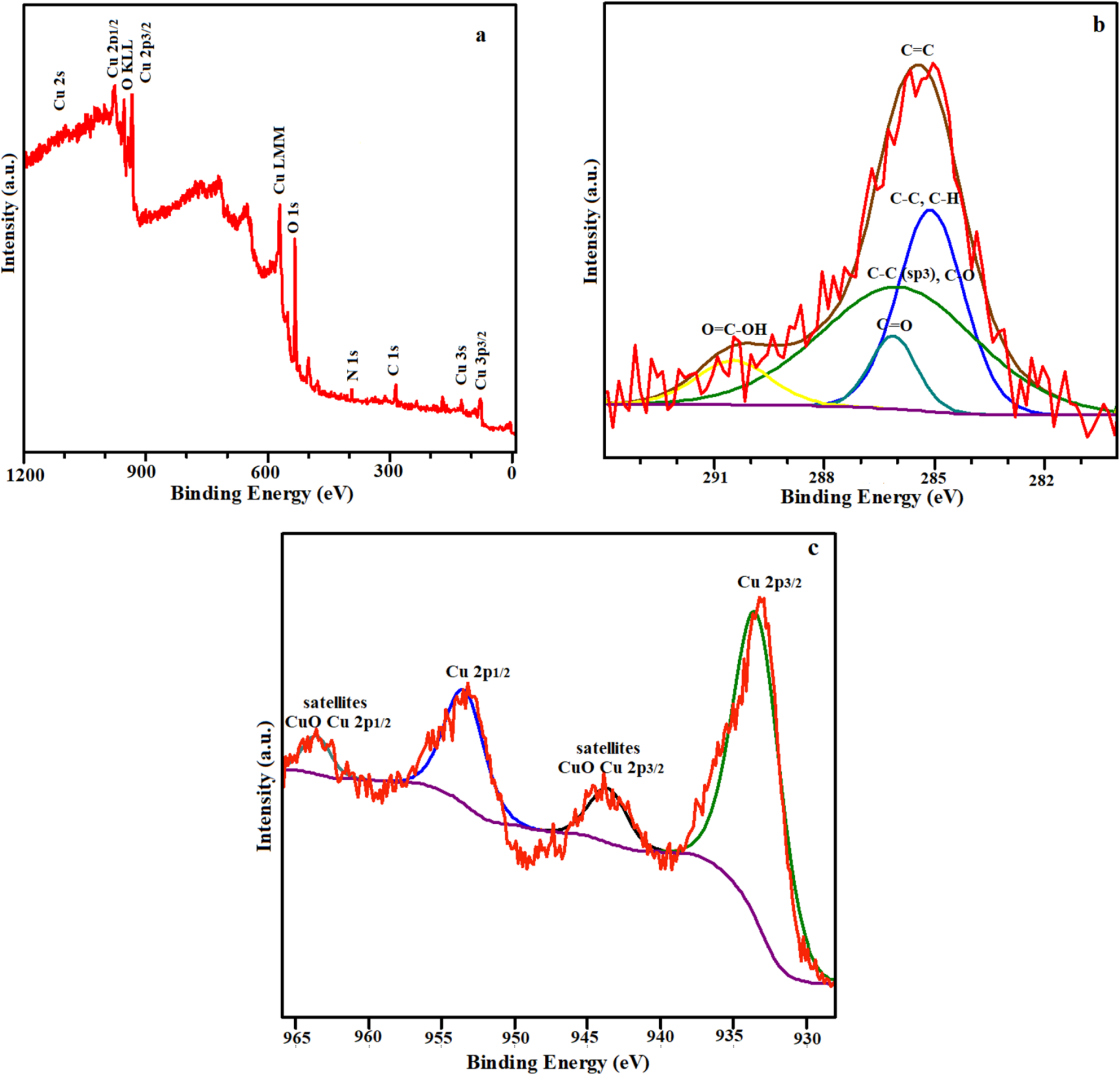
(**a**) Survey XPS spectrum of CuO/NCNO, High-resolution XPS spectrum of (**b**) C1s and (**c**) Cu 2p from CuO/NCNO.

To additional approve the chemical composition of the prepared nanoostructures, X-ray photoelectron spectroscopy (XPS) analysis was executed. A measured spectrum of the CuO/NCNO nanocomposite is shown in Fig. 3a, which demonste that C, Cu and O elements are detected and no other impurity is present^61^. Three distinct aspects that represent three various chemical states in carbon may be extracted from the high-resolution C 1s peak of CNOs in Fig. 3b: The graphitic structure of C=C is responsible for the peak at 284.5 eV, whereas C–C bonds are responsible for the peak at 285.3 eV. Furthermore, C-O is associated with large peaks at 286.9 eV. Furthermore, the existence of C=O is blamed for the peak at 289.8 eV^62^. The high-resolution XPS spectrum of Cu 2p is shown in Fig. 3c. The peak positions of Cu 2p1/2 and Cu 2p3/2 were 953.8 eV and 933.8 eV, respectively. The fitted peaks at 934.5 eV and 954.7 eV are relevanted to CuO. In addition, the two detectable shake-up satellite peaks further indicate the existence of CuO^63^. The results are in good agreement with those of XRD, which further confirms the successful fabrication of CuO/NCNO composite.

**Figure 4:**
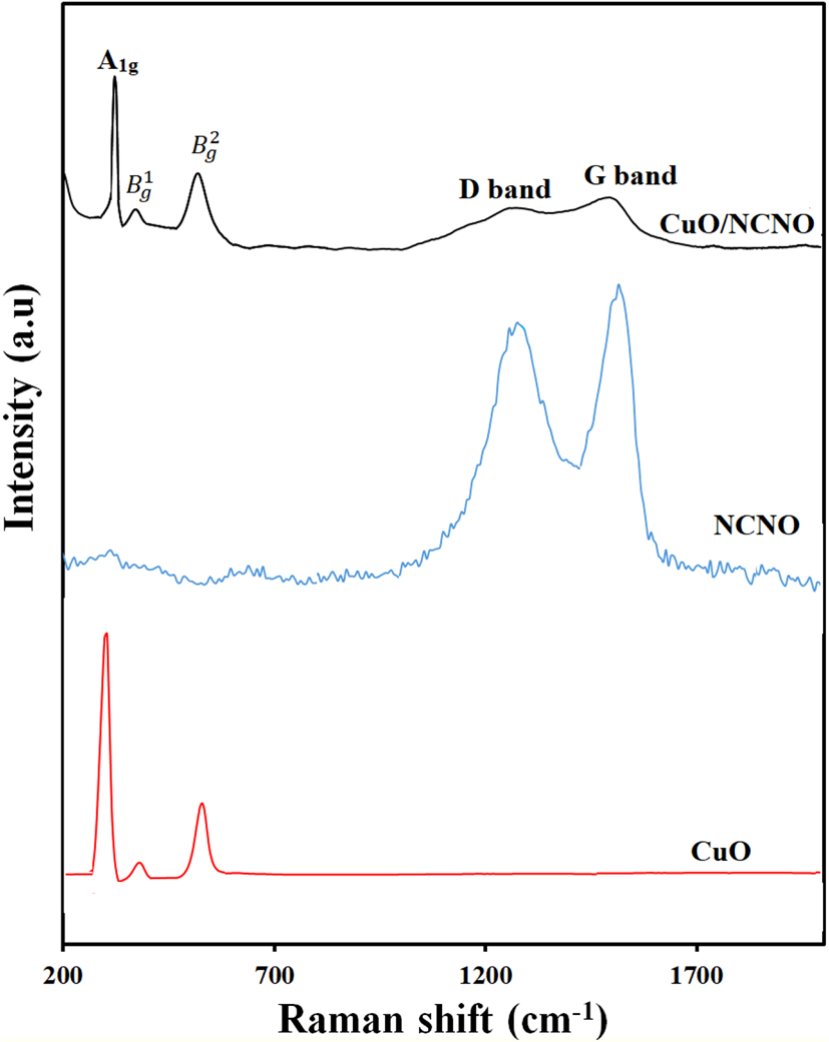
Raman spectra of CuO, NCNO and CuO/NCNO.

The Raman spectra of CuO, NCNO and CuO/NCNO are shown in Fig. 4. This figure shows two distinct peaks at 1350 and 1570 cm^−1^ for NCNO. The first peak at D band is related to structural defects, and the second peak at g band is related to E2g vibrations in aromatic carbons. Also, peaks related to CuO/NCNO can be seen in the Raman spectrum, which indicates the existence of NCNO in the CuO/NCNO nanocomposite. Other peaks observed in the Raman spectrum of CuO/NCNO nanocomposite correspond to those of the Raman spectrum of CuO. The observed peak is related to the monoclinic vibrations of the crystal structure, 291 cm^−1^, 337 cm^−1^, and 611 cm^−1^ are related to the A1g, ${B}_{g}^{1}$ and ${B}_{g}^{2}$, respectively.

**Figure 5:**
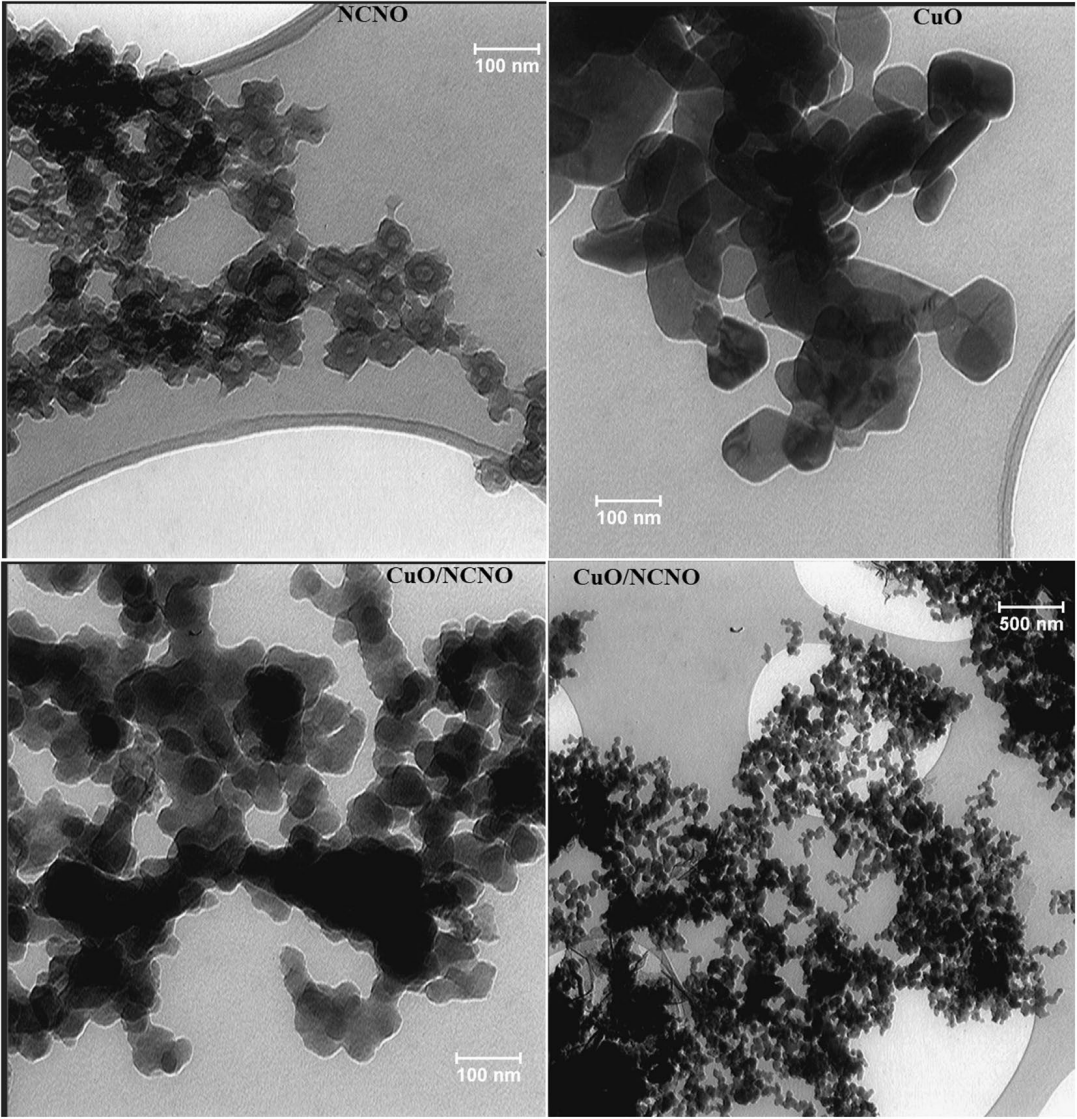
TEM imge nanomaterials.

TEM images of CuO, NCNO, and their nanocomposite are shown in Fig. 5. Tiny spherical nanoparticles are related to NCNO. Also, the images related to the flower-like CuO show the successful synthesis of this nanoparticle; the same morphology can be seen for CuO in the presence of NCNO.

### Investigation of supercapacitor properties of prepared electrodes

**Figure 6:**
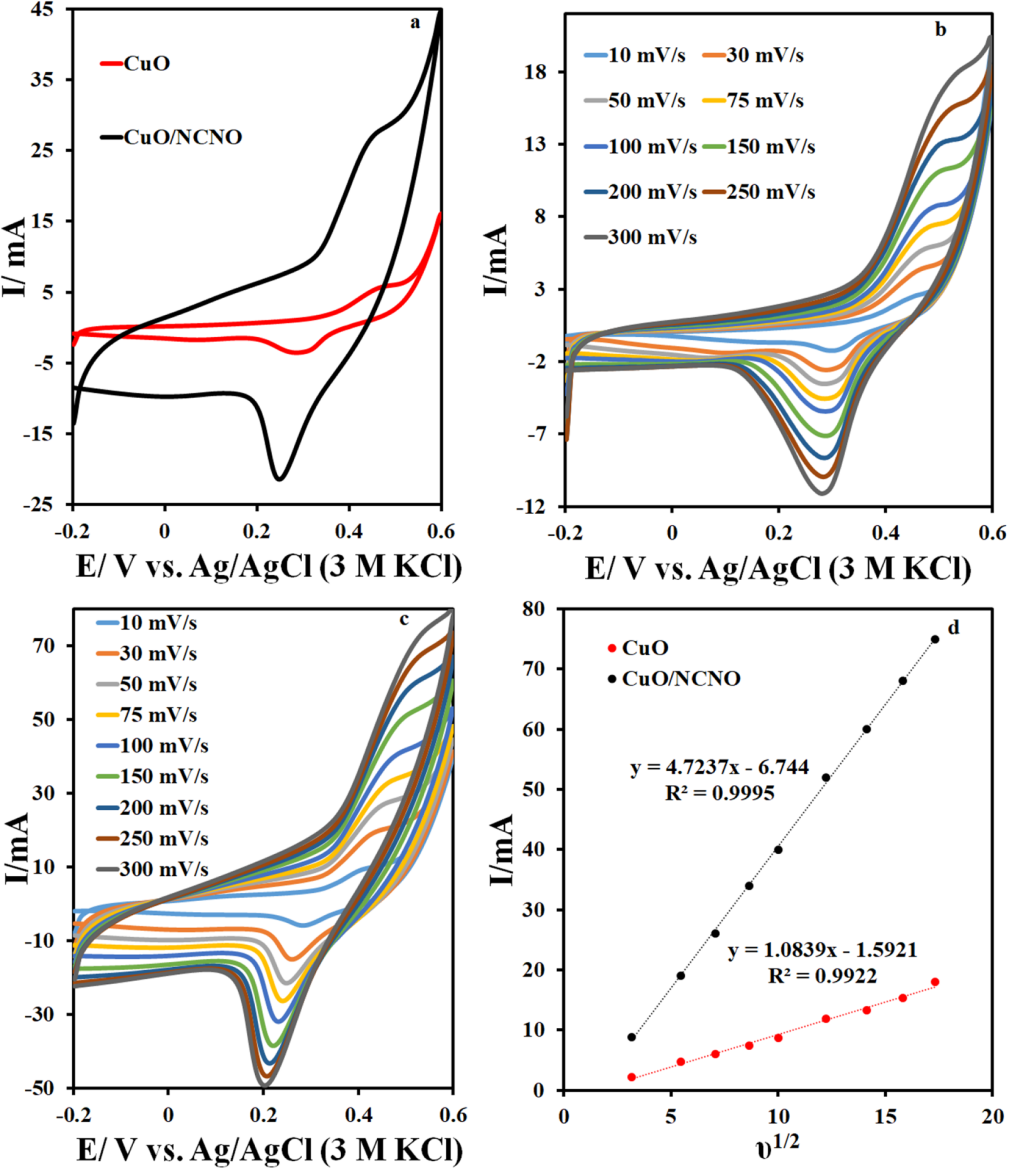
(**a**) CV curves of CuO and CuO/NCNO in potassium hydroxide at scan rate 50 mV.s, (**b**) CV curves of CuO at differn scan rats, (**c**) CV curves of CuO/NCNO at differnt scan rats and (**d**) I versus υ^1/2^.

The supercapacitor behavior of CuO and CuO/NCNO nanocomposite in potassium hydroxide solution was investigated by CV in a potential range of − 0.2–0.65 V at a scan rate of 50 mV/s. The experiments were performed in a three-electrode system, including the reference electrode (saturated Ag/ AgCl), the counter electrode (platinum wire), and the working electrode (CuO and CuO/NCNO). The cyclic voltammograms of CuO and CuO/NCNO are shown in Fig. 6a. The cyclic voltammogram of the CuO electrode shows a pair of oxidation and reduction peaks that correspond to the conversion of copper (I) to copper (II) and vice versa, however, its current is not very high, so it is a quasi-reversible reaction. A better reversibility reaction with a higher oxidation and reduction current for CuO is observed when the flower-like copper oxide is formed in the presence of NCNO. Also, the surface area under a curve of CuO/NCNO electrode is greater than that of the CuO electrode due to the high surface area of NCNO, which results from a synergistic effect of CNO and CuO components. The oxidation and reduction current and surface area under the curve are more for this electrode. The shape of CV curve of CuO/NCNO electrode is more significant than that of the copper oxide electrode, indicating the higher capacitance of CuO/NCNO electrode than CuO electrode. As the cyclic voltammograms of the CuO/NCNO electrode show, the area under the CV curve is high, and an increase in the current (in the anodic and cathodic sections) is observed due to the 3D structure containing high-conductivity carbon nanomaterials. In fact, the synergistic effect between CuO nanoparticles and NCNO is quite evident; as a result, the increase in the capacitance of the CuO/NCNO electrode is observed. The electrochemical reaction that occurred on the CuO/NCNO electrode during the electrochemical process is suggested as follows:

5 $${\text{CuO}} + {\text{H}}_{{2}} {\text{O + e}}^{ - } \leftrightarrow {\text{CuOH}}$$

6 $${\text{CuOH}} + {\text{OH}}^{ - } \leftrightarrow {\text{Cu(OH)}}_{{2}} {\text{ + e}}^{ - }$$

Cyclic voltammograms of CuO and CuO/NCNO electrodes at different scan rates are shown in Fig. 6b,c. According to these figures, the oxidation and reduction current increases gradually with increasing scan rate, indicating ideal capacitive behavior of this electrode. Also, shifting the anodic peaks and cathodic peaks toward the positive and the negative potentials, respectively, verify a quasi-reversible reaction for all the curves. The diagram of the current versus the square root of the scan rate is also displayed in Fig. 6d. There is a linear relationship between the oxidation peak currents and the square root of the scan rate for both CuO and CuO/NCNO electrodes, which shows that a quasi- reversible reaction has occurred on the surface of the electrode under the diffusion-controlled process. The voltammograms at various scan rates indicate that the Faraday current increases gradually as the scan rate increases, implying the ideal capacitive behavior of electrodes. Also, all cyclic voltammograms show a quasi-reversible process. As the voltammograms show, anodic peaks shift towards positive potentials, and cathodic peaks shift towards negative potentials as the scan rate increases, which is the main characteristic of pseudo-reversible reactions.

**Figure 7:**
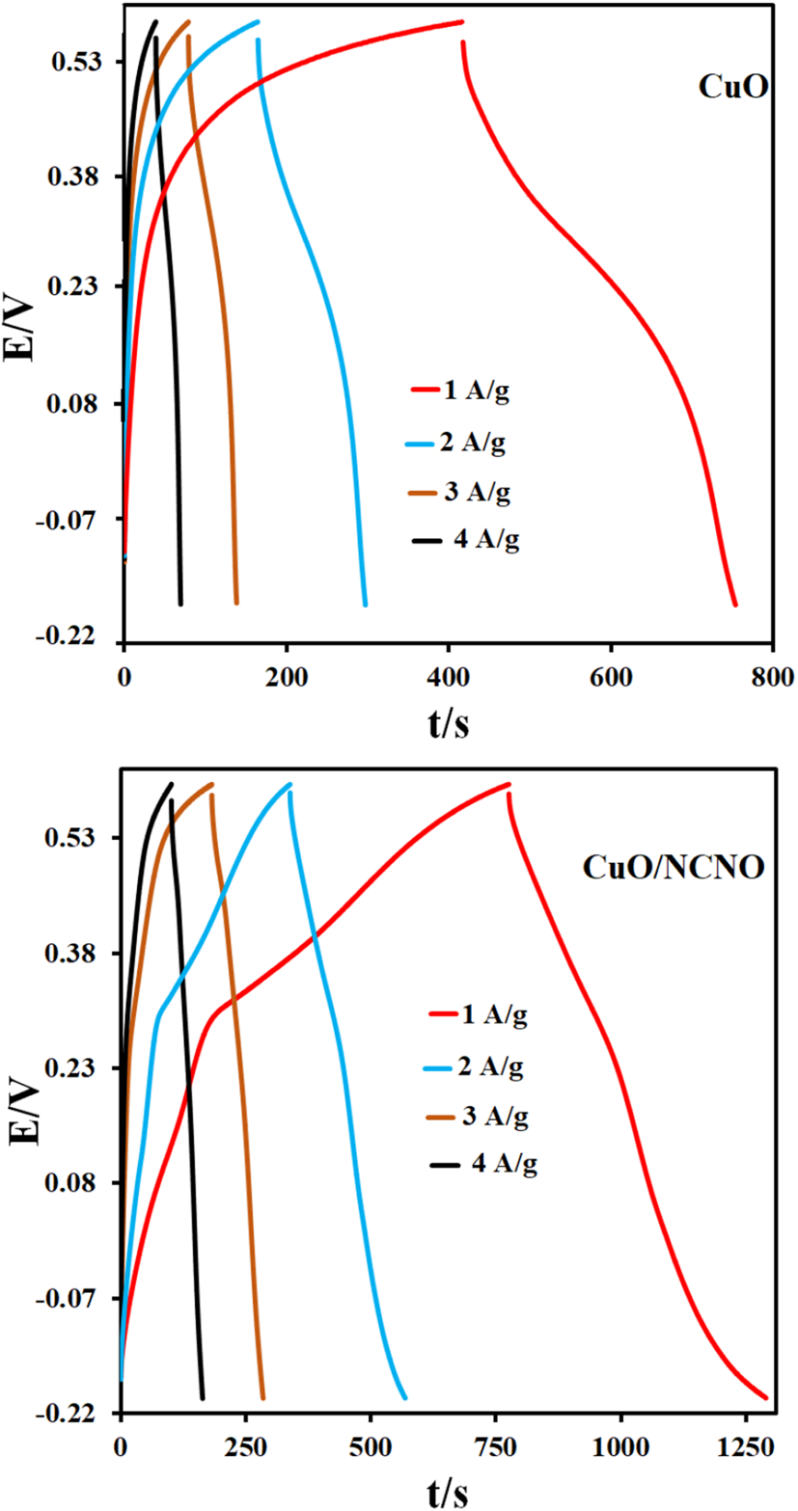
charge and discharge curves of CuO and CuO/NCNO.

The specific capacitances of CuO/NCNO and CuO electrodes were investigated in a three-electrode system with potassium hydroxide solution as an electrolyte and chronopotentiometry method (Fig. 7). The charge/discharge curve was obtained in the potential range of -0.2 to 0.6 V and the current density of 1, 2, 3, and 4 A/g. As the current density decreases, the charge/discharge time becomes longer. Figure 7 shows the charge and discharge curves of CuO and CuO/NCNO electrodes. The obtained capacitances for the CuO at current densities of 1, 2, 3, and 4 A/g are 441.25, 367.5, 221.25, and 155 F/g; however, the capacitances values for CuO/NCNO electrodes at the mentioned current densities are 673.75, 572.5, 382.5 and 315 F/g, respectively. As it is known, the estimated specific capacitance decreases with increasing current density, which indicates that the electrode materials have little effect on electrochemical reactions.

**Figure 8:**
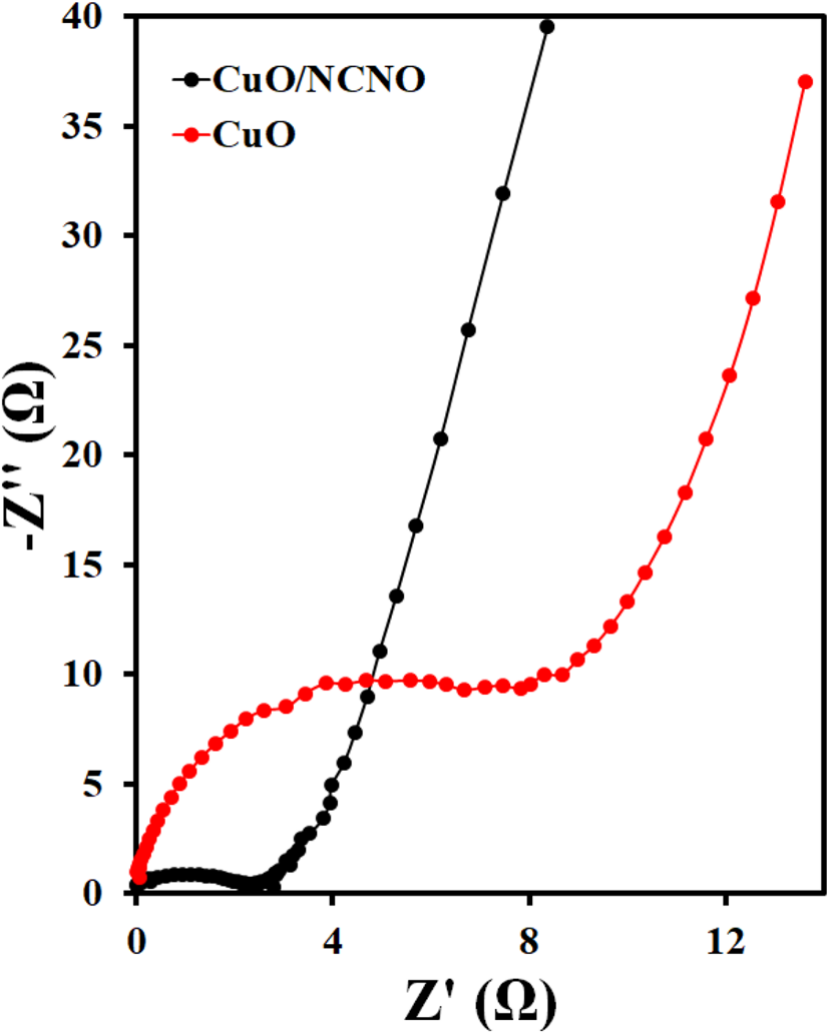
Nyquist plots of CuO and CuO/NCNO.

The EIS curves of the mentioned electrodes was obtained in a potassium hydroxide solution to check the resistance and conductivity of electrodes. The results are shown in Fig. 8. As the results also indicate, the charge transfer resistance of CuO/NCNO is lower than that of CuO electrode, which suggests that the electron transfer rate on the surface of this electrode is higher than that of the CuO electrode; therefore, the conductivity and capacitance of the electrode are higher. A comparison of the performance of the capacitive electrodes based on CuO and CuO/NCNO is reported in Table 1. The capacitance in this work is comparable and even superior than other electrodes due to the synergistic effect of CuO and NCNO. The CuO/NCNO capacitive electrode has a higher surface area and more active sites for the diffusion of the ions and Faraday reactions; therefore, better electrical conductivity is obtained at a more suitable charging time.

**Table 1:** A comparison of the performance of the capacitive electrodes based on CuO.

Electrode	Electrolyte	Morphology	Current density (A.g)	specific capacitance (F.g)	Refs.
RGO/CuO/Cu_2_O	6 MKOH	Spherical nanoparticles	1	173	^64^
PPD/CuO/RGO	6 M KOH	Spherical nanoparticles	1	512	^65^
CuO/CuCo_2_O_4_	3 M KOH	Nanorods	1	443	^66^
RGO/CuO	1 M KOH	Honeycomb-like	0.2	188	^67^
Cu-CuO/Cu_2_O	6 M KOH	Nanowires	1	85	^68^
CuO/g-C_3_N_4_	3 M KOH	Spherical nanoparticles	2	384	^69^
CuO/NCNO	3 MKOH	Flower like	1	673.75	This work

The greater electrochemical activity results from the synergistic impact of the double composite, which has high surface area providing more active sites, stronger electronic conductivity that offering fast charge transfer, and enhanced electrochemical performance of the CuO/NCNO electrode. Additionally, it is taken into account that the cyclic stability of electrode is a crucial element for practical applications.

**Figure 9:**
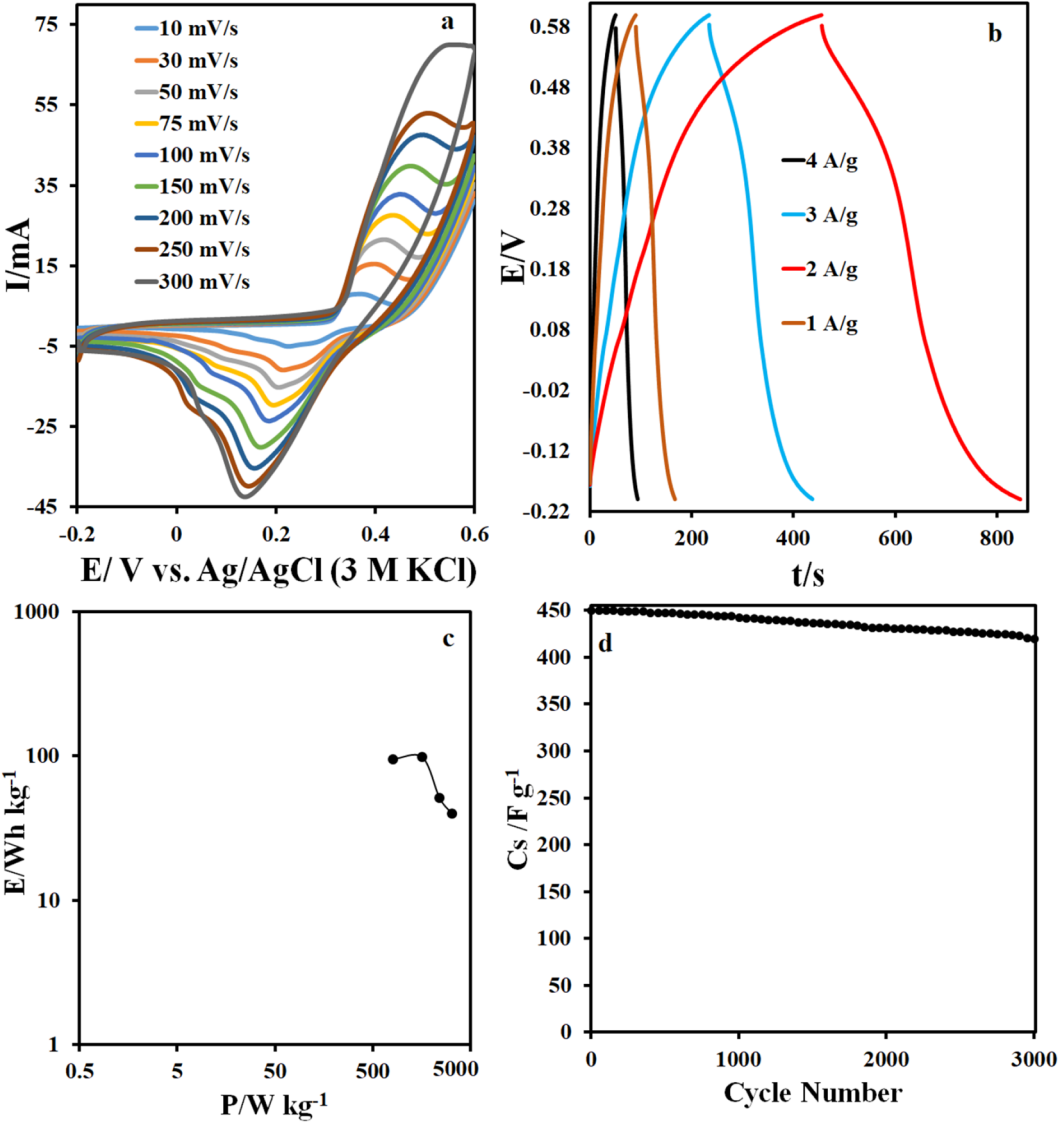
(**a**) CV curves of CuO/NCNO symmetrical system, (**b**) charge and discharge curves, (**c**) Ragone plot and (**d**) stability plot.

**Figure 10:**
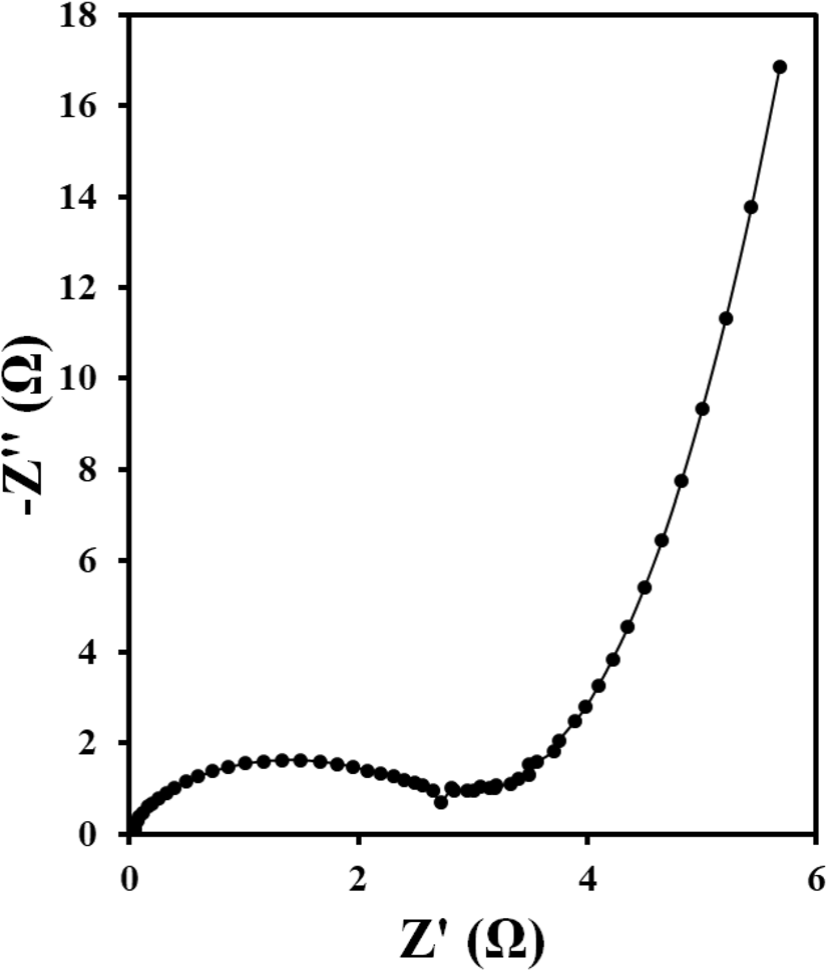
Nyquist plot of CuO and CuO/NCNO.

The supercapacitor behavior of a symmetrical CuO/NCNO electrode system in a potassium hydroxide solution was investigated by the CV method. Cyclic voltammograms of the CuO/NCNO electrode in Fig. 9a show a pair of oxidation/reduction peaks that appears for this electrode, which is shifted by increasing the scan rate. In fact, a quasi-reversible behavior in the potential region of − 0.2–0.6 V is related to the oxidation/reduction reaction of copper with a favorable oxidation/reduction current in the presence of NCNO. Electrode capacitance, power, and energy density of CuO/NCNO electrode were investigated using chronopotentiometry method. The obtained capacitances for CuO/NCNO electrode in the current density of 1, 2, 3, and 4 A/g are 1072.5, 1015, 577.5, and 450 F/g, respectively (Fig. 9b). Also, the maximum power and energy density for this electrode are 98 W/kg and 3200 Wh/kg, at the current density of 1 and 4 A/g, respectively. The Ragone plot, Fig. 9c, shows that good quasi-capacitive behavior can be considered for this electrode due to the appropriate power and energy density. To study the stability of the CuO/NCNO electrode, 3000 consecutive charge/discharge cycles were recorded at a current density of 4 A/g. The results showed that the capacitance change of the CuO/NCNO electrode in the final cycle compared to the first cycle is 4%, so it can be conclude that the CuO/NCNO capacitive electrode has acceptable stability (Fig. 9d). Also, the charge transfer resistance in the potassium hydroxide solution based on the Nyquist diagram is 3 Ω indicating the excellent conductivity of this electrode (Fig. 10).

## Conclusion

A supercapacitor electrode with suitable performance characteristics was prepared using flower-like NCNO and CuO. The conductivity of flower-like CuO electrode was improved due to the high surface area of CuO. This supercapacitor also provides a specific capacitance of 455 F/g (in a symmetrical system at 4 A/g) and a good stability after 3000 cycles. Therefore, the supercapacitor electrode obtained in this work as an energy storage device can provide acceptable results. The mentioned electrode is prepared without additives, and its preparation is more accessible than similar ones. As a result, it can be used as a low-cost, high-performance electrode in addition to the desirable supercapacitor characteristics.

## Data Availability

The data used to support the findings of this study are available from the corresponding authors upon request.
